# Transcriptional control in embryonic Drosophila midline guidance assessed through a whole genome approach

**DOI:** 10.1186/1471-2202-8-59

**Published:** 2007-07-31

**Authors:** Tiago R Magalhães, Jessica Palmer, Pavel Tomancak, Katherine S Pollard

**Affiliations:** 1 Programa Gulbenkian Doutoramento Biologia e Medicina, Centro Neurociências, Universidade de Coimbra, 3000 – Coimbra, Portugal; 2 Lewis-Clark State College, 500 8th Avenue, Lewiston, ID 83501, USA; 3 Max-Planck-Institute of Molecular Cell Biology and Genetics, Dresden, Germany; 4 UC Davis Genome Center & Department of Statistics, University of California, Davis, CA, 95616, USA

## Abstract

**Background:**

During the development of the Drosophila central nervous system the process of midline crossing is orchestrated by a number of guidance receptors and ligands. Many key axon guidance molecules have been identified in both invertebrates and vertebrates, but the transcriptional regulation of growth cone guidance remains largely unknown. It is established that translational regulation plays a role in midline crossing, and there are indications that transcriptional regulation is also involved. To investigate this issue, we conducted a genome-wide study of transcription in Drosophila embryos using wild type and a number of well-characterized Drosophila guidance mutants and transgenics. We also analyzed a previously published microarray time course of Drosophila embryonic development with an axon guidance focus.

**Results:**

Using *hopach*, a novel clustering method which is well suited to microarray data analysis, we identified groups of genes with similar expression patterns across guidance mutants and transgenics. We then systematically characterized the resulting clusters with respect to their relevance to axon guidance using two complementary controlled vocabularies: the Gene Ontology (GO) and anatomical annotations of the Atlas of Pattern of Gene Expression (APoGE) in situ hybridization database. The analysis indicates that regulation of gene expression does play a role in the process of axon guidance in Drosophila. We also find a strong link between axon guidance and hemocyte migration, a result that agrees with mounting evidence that axon guidance molecules are co-opted in vertebrate vascularization. Cell cyclin activity in the context of axon guidance is also suggested from our array data. RNA and protein expression patterns of cell cyclins in axon guidance mutants and transgenics support this possible link.

**Conclusion:**

This study provides important insights into the regulation of axon guidance in vivo.

## Background

The process by which axons cross the midline during development of the Central Nervous System (CNS) in Drosophila is of great interest and has been the focus of much scientific research [1,2]. After neuroblast delamination and cell fate decisions, axons undergo a journey that will eventually wire them to the appropriate targets. This journey includes a series of steps, one of which is the decision to cross (or not cross) the midline. Four key regulators of midline crossing are known: slit, robo, robo2 and comm. Growth cones in which the robo and robo2 receptors are present at the membrane sense the presence of the slit ligand and do not cross the midline [3,4]. Conversely, growth cones without robo and robo2 at the membrane are not able to respond to the repulsive cue of slit and do cross the midline. robo levels at the growth cone membrane are regulated by comm.

A major question in midline crossing is how regulation occurs through gene expression, i.e., to what extent is transcription relevant in the control of growth cone pathways. It is well established that rapid local translation does occur [5,6]. Some literature also indicates that transcription is relevant [7-9]. We designed this study of transcription during CNS development to i) find relevant genes in the axon guidance process, ii) elucidate the role of transcription regulation during midline crossing in Drosophila, and iii) determine whether any upstream changes are visible in axon guidance mutants. Our analysis uses two whole-genome microarray data sets: (1) *axon guidance *– Drosophila embryos, stage 15, of axon guidance Gain Of Function (GOF) and Loss Of Function (LOF) mutants, and (2) *time course *– a developmental wild type time course, consisting of 12 non-overlapping 1 hour collection points (previously published [10]). The axon guidance (AG) data was collected as part of this study and is published here for the first time. We have thoroughly reanalyzed the previously published time course (TC) data.

To identify clusters of co-expressed genes in each data set, we employ the *hopach *clustering methodology [11]. Clustering algorithms have been successfully applied to microarray studies, but the systematic analysis of clustering output remains a major challenge. A significant obstacle is the lack of reproducibility, or even assessment of variability, of clustering results. Clustering output can vary dramatically from one repetition of an experiment to another, particularly with the small sample sizes typical in microarray experiments. The R statistical package *hopach *[11] uses a bootstrap resampling method to assess cluster variability, producing a measure of membership of each gene in every cluster. For this study, we adapted *hopach *to use these cluster membership values to assign genes to clusters, while allowing genes to belong to more than one cluster – a type of fuzzy clustering. This modification improves reproducibility and results in cluster output that is suitable for functional characterization. *hopach *is freely available and is easily used by biologists, providing a statistically sound framework for analysis of microarray data.

We characterize gene clusters with two complementary sources of annotation, the Gene Ontology (GO) [12], and the Atlas of Pattern of Gene Expression (APoGE), a component of the Berkeley Drosophila Genome Project [10]. APoGE provides pictures of the expression pattern of each gene in the Drosophila embryo and a description of the anatomical structures in which the gene is expressed. A controlled vocabulary is used by individual curators (Amy Beaton and Volker Hartenstein), experts who assure consistency in each call. The goals and scope of APoGE are described in [10]. Its use for characterization of microarray data has never been explored. Together, GO and APoGE provide a rich, multi-dimensional vocabulary for annotating *hopach *clusters. In this study, we analyze clusters in which genes with terms related to axon guidance are significantly over represented. Querying the APoGE in situ database for genes expressed with specific spatial and temporal patterns provides a means to further explore the axon guidance clusters.

Applying this approach, we identify striking changes in gene expression across GOF/LOF, as well as groups of axon guidance genes co-expressed during wild type development. These findings suggest that regulation of gene expression does in fact play a role in midline crossing. In addition, our analysis indicates that axon guidance genes may be involved in the process of hemocyte migration, and that cell cyclins are active during midline crossing. These observations are confirmed through independent RNA and protein expression studies.

## Results and discussion

### Transcriptional Control in Axon Guidance Mutants

**Figure 1:**
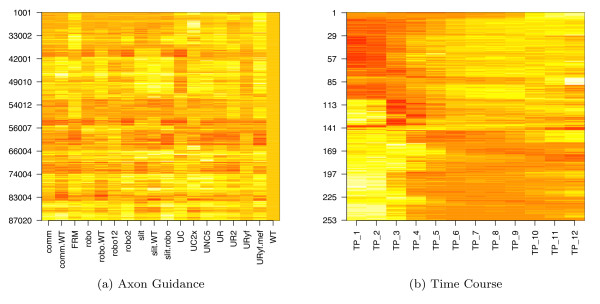
**Medoid Heat Maps of Axon Guidance and Time Course**. Heat map of the medoids identified in (1a) axon guidance and (1b) developmental time course experiments. The medoid is a single gene from each cluster, selected by *hopach *as the cluster profile because its pattern of expression is the most representative of that cluster. Medoid plots provide a global overview of the clustering results. The order of clusters in these plots is uniquely determined by the *hopach *algorithm. Expression relative to the control is shown on a red to yellow color scale, with red indicating suppressed and yellow indicating induced expression. The number of genes represented by each profile (i.e., row) is variable from cluster to cluster. (1a) Medoids for the axon guidance data. There are distinct regions of clusters, for instance the initial 37 clusters are similar with stronger expression for the gain of function FRM and UR.mef. A distinct region follows, in which there is a significant decrease in the mutants robo, robo2, slit.robo and the transgenic UC2x. (1b) Medoids for the developmental time course. The initial clusters show only late expression with the subsequent clusters showing earlier and earlier expression. Note: the size of each cluster is not represented in this plot. For instance Cluster TC139 has 6626 genes -almost half of all microarray probes are represented by one single row.

Our *hopach *analysis of gene expression profiles from 17 Drosophila axon guidance mutants identified 517 clusters of median size 27 (range 1 to 821). Figure 1 shows the profiles of these clusters ordered by the *hopach *algorithm so that clusters close to each other contain genes with similar expression profiles. If regulation during the course of axon guidance occurs mainly through protein translation, the changes observed in a microarray study will not yield coherent groups of genes with known function in axon guidance. Yet, we found several clusters significantly enriched for axon guidance genes.

We ranked clusters based on their association with relevant terms from GO ("axon guidance") and APoGE ("ventral nerve cord", "embryonic brain", and "ventral midline") as described in the Methods. The top four clusters for each term are shown in Table 1. Cluster AG132 ranks first for "axon guidance", "embryonic brain" and "ventral midline". It is the cluster with the highest ranks in both the GO and the APoGE vocabularies. This cluster contains a number of genes well known from the axon guidance literature, such as dock, plexB, Cam, CadN, caps, spen, Fs(2)Ket, Con, RhoGAP19D, Rac2, and shot. AG164 is another cluster that also contains relevant genes in axon guidance mechanisms, such as trio, WASp, Nedd4, robo3, MICAL, RhoGAPp190, rhoGAP88C, RhoGAP18B, lola, side, and Fmr1 [2]. A full list of genes in all of the top ranking clusters and a more detailed exploration of each cluster are available in the Supplementary Materials(Additional files 1,2,3,4).

**Table 1:** Significant Clusters for Relevant Controlled Vocabulary Terms in Axon Guidance Data

			Significant Clusters for Each Term in AG Data								
Axon Guidance			Emb Brain			VNC			Ventral Midline		

Cluster	ClSizes	Pvalue	Cl	Size	Pvalue	Cl	Size	Pvalue	Cl	Size	Pvalue

AG132	162	2.4e-05	AG132	162	2.19e-07	AG148	71	3.04e-05	AG132	162	1.29e-08
AG164	281	3.12e-05	AG091	48	8.74e-05	AG482	19	7.78e-05	AG147	31	3.41e-05
AG131	99	0.000203	AG147	31	9.1e-05	AG210	42	0.000203	AG302	55	6.98e-05
AG172	55	0.000274	AG146	264	0.00012	AG477	55	0.000789	AG131	99	0.000119

**Figure 2:**
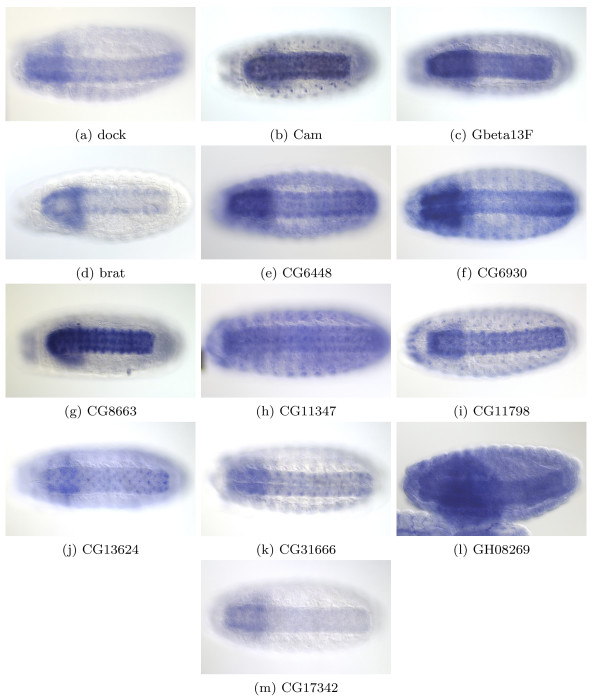
**Genes Present in APoGE for Cluster AG132**. Genes present in APoGE for Cluster AG132 show a similar pattern in embryonic brain and ventral nerve cord. All embryos are viewed ventrally. (2a) dock has a key role in axon guidance [47]. (2b) Cam has been implicated in photoreceptor light termination, muscle synapses and midline crossing [48]. (2c) Gbeta13F mutants have neuroblasts defects [49]. (2d) Brat is reported as involved in the regulation of cellular rRNA. Genes (2f) to (2m) are genes of unknown function with a similar pattern of expression.

Querying APoGE, we identified a large subset of genes in Cluster AG132 which are expressed in the commissures and midline (Figure 2). Several genes of unknown function (CG6448, CG6930, nrv3, CG11347, CG11798, CG13624, and CG31666) share this distinctive pattern of expression with the known genes dock, gBeta, brat and Cam. This similarity provides some insight into the functions of the less well characterized genes and suggests their involvement in axon guidance.

### Hemocyte Migration

Querying APoGE for Cluster AG164 reveals genes with hemocyte expression, including Dgk, pyd, 103E12, CG8780, CG11100, CG18549 and CG32354. Hemocytes are Drosophila blood cells, which migrate during embryonic development [13], with the direct involvement of the Vascular Endothelial Growth Factor (VEGF) pathway [14]. Hemocytes are relevant in CNS development [15], and it is known that mutants for Pvr, a key molecule in the VEGF pathway, show a longitudinal phenotype and CNS condensation defects [16].

**Figure 3:**
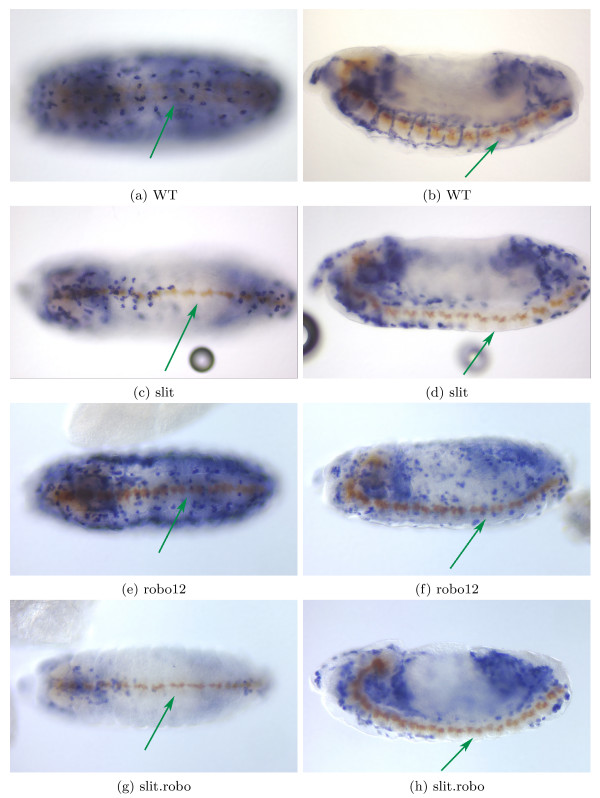
**Hemocyte Location in Axon Guidance Mutants**. In blue, RNA in situ hybridization showing expression for CG25C, a hemocyte probe. The CNS scaffold (brown) is stained with the BP102 antibody, and indicates the CNS phenotype. The ladder-like WT CNS is visible in WT; the collapsed phenotype is observed in slit, robo12 and slit.robo embryos. (3a), (3c), (3e) and (3g) are ventral views; (3b), (3d), (3g) and (3h) are lateral views. Green arrows indicates the middle segments where in some mutants the hemocytes are absent. (3a) and (3b) are WT embryos where the hemocytes are present ventrally along the entire anterior-posterior axis. (3c) and (3d) are homozygous slit embryos where the hemocytes are not present in the middle section of the embryo. (3e) and (3f) are double mutants for robo and robo2 (robo12). The collapsed midline phenotype is similar to slit and slit.robo mutants, and yet, contrary to slit and slit.robo, the hemocytes are present throughout the whole embryo. (3g) and (3h) are embryos double mutant for robo and slit. They are similar to the slit homozygous mutants in that the hemocytes are not present in the middle section of the embryo.

The microarray data indicates that genes expressed in hemocytes are significantly upregulated in slit and robo12, but not in slit.robo. This pattern is intriguing, because the axon guidance phenotype (all axons collapsed at the midline) is similar in the three mutants. Motivated by this observation, we investigated whether hemocytes show any spatial defects in mutants for the slit ligand (slit), the double mutant for slit's receptors robo1 and robo2 (robo12), and the double mutant for slit and robo (slit.robo). Figure 3 is representative of the data we observe using two diffent probes (fray and CG25C); the CNS scaffold is visible by staining with the antibody BP102. The robo12, slit and slit.robo embryos have the same midline CNS phenotype, with all axons collapsed at the midline (highlighted with the green arrow). Hemocyte expression, however, differs between the mutants. The hemocytes are absent from the middle segments in slit and slit.robo embryos (3c, 3d, 3g and 3h), whereas the defect is not observed in the robo12 embryos (3e and 3f). This raises the speculation that slit might be part of some repellent mechanism for hemocytes, with robo assuming a different role than in axon guidance. From the in situ hybridization data it is not possible to determine whether genes expressed in hemocytes are up regulated in slit and slit.robo mutants. The hemocytes are absent in the middle segments, but expression in hemocytes in other segments could be higher, leading to a higher overall expression in whole embryos. Nevertheless, we can conclude that the slit and slit.robo have the same pattern, different from robo12, as the microarray data suggested.

### Transcriptional Control in Time Course

Applying *hopach *to the developmental data set first published in [10] results in 253 clusters. One of these (cluster TC139) contains 6626 non-differentially expressed genes (with a similar level of expression in all developmental stages). This result illustrates the ability of the *hopach *algorithm to place low-variability genes in a single large cluster, while forming distinct (much smaller) clusters for other patterns.

The key axon guidance genes slit, comm, comm2 and robo2 belong to clusters significantly enriched for the GO term "axon guidance" (Table 2). The genes comm, comm2 and robo2 are all present in Cluster TC91, which is a small cluster (15 members) with a pattern of expression characterized by a peak around stages 4 to 9. Comm is known to be transiently expressed in the commissural axons and midline glia, simultaneously with robo [17,18]. However, the similarity in expression for comm, comm2, and robo2 throughout the whole 18 hours of development has never been reported. Given the exceptional similarity between the expression of these three genes it would be interesting to explore if they are controlled by the same upstream mechanism, or whether they control a common downstream mechanism. Included in the adjacent clusters (TC90 to TC97), hence with a very similar pattern of expression, there are other important axon guidance genes (RhoGEF4, neur, and RhoGEF3) and many genes involved in Notch signaling (E(spl), neur, Brd, m4, HLHm5, HLHmgamma, Dl, bib and malpha). Notch involvement in axon guidance has been previously reported [19]. The genes spi, hh, argos, gsb, Dl, slp2, siz, and ems are also in these clusters and have documented phenotypes in axon guidance or the CNS scaffold.

**Table 2:** Significant Clusters for Relevant Controlled Vocabulary Terms in Time Course Data

			Significant Clusters for Each Term in TC Data								
Axon Guidance			Emb Brain			VNC			Ventral Midline		

Cluster	ClSizes	Pvalue	Cl	Size	Pvalue	Cl	Size	Pvalue	Cl	Size	Pvalue

TC52	21	5.22e-06	TC141	109	1.21e-15	TC10	13	0.000303	TC140	146	1.43e-16
TC91	15	2.77e-05	TC140	146	1.14e-11	TC07	14	0.000545	TC141	109	1.99e-12
TC27	12	3.68e-05	TC142	126	5.49e-11	TC97	17	0.00133	TC142	126	5.59e-11
TC54	41	4.3e-05	TC97	17	0.000267	TC47	20	0.0016	TC10	13	0.000171

A query of APoGE for genes in Clusters TC90 to TC97 with midline expression similar to comm, comm2 and robo2, yielded argos. The gene argos has been shown to be relevant in axon guidance in the visual system [20], which further strengthens the possibility of a role for argos in midline axon guidance. Sulfated (Sulf1) is expressed in cells that appear to be adjacent to comm2 and robo2 expressing cells. Sulf1 has not been previously implicated in axon guidance. It is our future project to investigate whether either argos or Sulf1 has a genetic interaction with comm, comm2 and robo2.

### Cell Cyclins

Time course clusters TC140, TC141 and TC142 are the most relevant clusters for the APoGE terms ("embryonic brain", "ventral nerve cord", "ventral midline"). They do not contain axon guidance genes, but they have many cyclins, including CycA, polo, cdc2, CycB3 CycE and ago. Cell cyclins are involved in cell division and in CNS development, playing an important role in neuroblast division. However, a role for cyclins has not been reported in post-mitotic neurons, the type of neurons involved in axon guidance. It is known that CycA and CycE mutants have severe defects in the commissures and longitudinal axon tracts of the CNS [21,22], and Cdk5 controls some aspects of axon patterning in vivo [23]. The cyclins CycA, cdc2 and CycB3 genetically interact with each other [24,25] and are important in the earlier stages of intense cell division [26]. Agreeing with this role, the TC data shows high expression early in development, but suggests other functions at later stages because i) there is sustained expression well past the intense initial cell divisions, and ii) the APoGE images show a similar pattern of expression for all cyclins for later stages.

#### Co-expression of Cell Cyclins in Axon Guidance and Time Course Data

**Figure 4:**
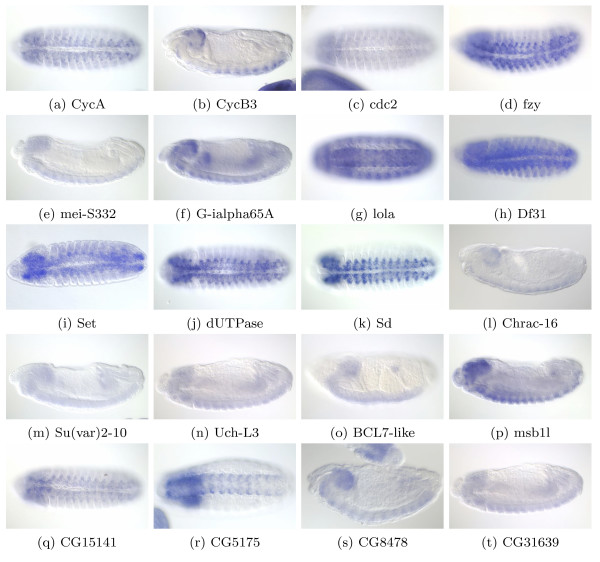
**RNA Expression in APoGE for Common Genes in Axon Guidance and Time Course Relevant Clusters**. (4a), (4c), (4d), (4g), (4h), (4i), (4j), (4k), (4q), (4r), ventral view. (4b), (4f), (4l), (4m), (4n), (4o), (4p), (4s) and (4t), lateral view. (4a) CycA is a cell cyclin whose mutants have severe defects in the commissures and longitudinal axon tracts of the CNS [21]. (4b) CycB3 is a cell cyclin, required for spindle organization. (4c) cdc2 is a cell cyclin; cdc2 mutants have CNS defects [50]. (4d) fizzy is involved in cell cycle and mutants result in degeneration of CNS and absence of PNS. (4e) mei-S332 is a gene involved in meiosis and mitosis. (4f) G-ialpha65A is involved in mitosis and is also involved in CNS development. (4g) lola is a transcription factor required for axon guidance. (4h) Df31 is involved in chromatin remodelling. (4i) Set is involved in DNA replication with reported cyclin binding characteristics. (4j) dUTPase is involved in nucleic acid metabolism. (4k) scalloped (sd) is involved in wing development and in CNS development. (4l) Chrac-16 is a member of the chromatin accessibility complex. (4m) Su(var)2–10 is involved in chromosome organization and biogenesis. (4n) Uch-L3 has been reported as a member of the regulatory complex of the 26 S Proteasome. (4o) BCL7-like and (4p) msb1l are genes for which little is known. (4q) CG15141, (4r) CG5175, (4s) CG8478, and (4t) CG31639 are genes with no functional information available but with the cell cyclin pattern of expression.

Interestingly, we also identify expression changes in cyclins in the AG data set. Clusters AG1 to AG93 are a group of cell cyclin rich clusters that contain the cyclins smg, polo, CycB3, swa, pim, cdc2, twe, CycB, and fzy. Table 3 shows the percentage of genes present in APoGE with cell cyclin-like expression in each data set separately and simultaneously in both of them. We reason that genes belonging simultaneously to the two independent data sets' cell cyclin rich cluster space have a higher probability of being a cell cyclin or cell cyclin related gene. Figure 4 depicts the genes from this common cluster space with cyclin-like expression. There are three types of genes in this set: i) genes with a role as cyclins (CybB3, cdc2, CycA, fzy, mei-S332, G-ialpha65A, and Set), ii) genes with no known role as cyclins (lola, Df31, Set, UTPase, Sd, msf11, Chrac16, Su(var)2–10, and Uch-L3), and iii) genes with little known information (CG15141, CG5175, CG8478, and CG31639). We plan to research the proposed role of the latter two groups of genes in the context of axon guidance.

**Table 3:** Cell Cyclin Expression in Axon Guidance and Time Course

Cell Cyclin Expression in Axon Guidance and Time Course			
Expression	Axon Guidance AG1-AG93 (%)	Time Course TC140-TC142 (%)	Overlap (%)

cell cyclin like	25	44	50
not cell cyclin like	14	13	17
undefined	61	43	33

	*n *= 102	151	30

#### Cell Cyclin Protein and RNA Expression in Axon Guidance Mutants and Transgenics

**Figure 5:**
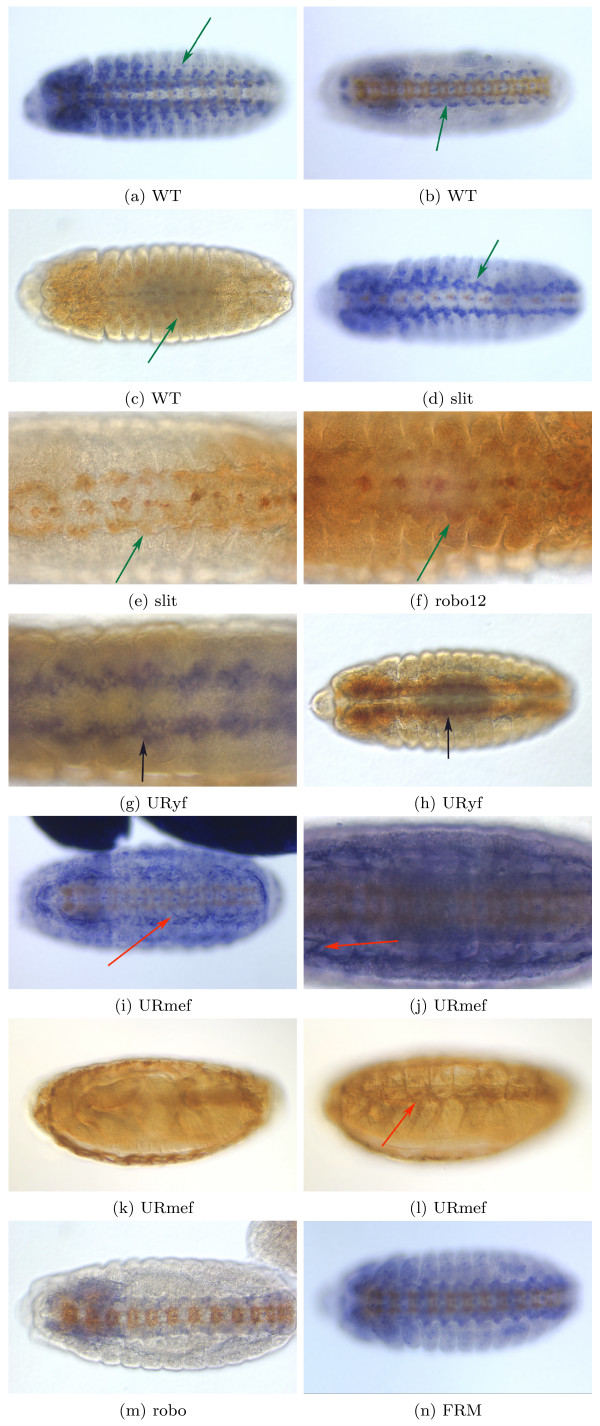
**RNA and Protein Cell cyclin Expression in the Axon Guidance Mutants and Transgenics**. All embryos are shown ventrally. (5a), (5c), (5e), (5f), (5g), (5i), and (5l) are mRNA expression patterns. (5b), (5d), (5h), (5j), and (5k) are protein expression patterns. (5a) CycA RNA expression (blue) in WT embryos, stage 13. The scaffold of the CNS is stained with antibody BP102 (brown). Cells expressing cyclins are visible on the ventral surface in the middle of the segmental commissures of the VNC. (5b) CycA protein staining in WT embryo, showing a similar pattern to the CycA RNA expression. (5c) CycB RNA expression in UR.yf embryos. There is a large increase in cyclin expression and cyclin-expressing cells are away from the midline (black arrow). There are no cyclin expressing cells at the midline. (5d) CycB protein staining in UR.yf embryos (black arrow). The pattern is similar to the RNA expression. (5e). CycB3 RNA expression in FRM embryos. There is an increase of cyclin expression. (5f) and (5g) CycB RNA expression for embryos with robo overexpressed in the muscles (UR.mef); an increase is seen in the muscles (red arrow), with the pleural external oblique muscles clearly visible in (5f). (5h) Cdc2 protein staining in UR.mef embryos. The staining is very clear in the muscles (red arrow) showing cyclin expression on the muscles, whereas in the WT embryos there is no such expression. (5i) CycA RNA expression in a slit embryo. The cyclin expressing cells are present on a narrow region outside of the VNC, compared to their central and lateral location in the WT (compare regions highlighted with the green arrow); staining at the midline cells does not change. (5j) The midline cells express CycB. In a wt embryo, additional cyclin-expressing cells are also visible in the area abutting the midline. However, in the slit embryo, cyclin-expressing cells are not visible in this area, instead appearing pushed outside the VNC. Also the more lateral cells are absent in the slit embryo. (5k) CycB protein staining of a robo12 embryo. As in the slit embryo (5j), cyclin expressing cells are absent from the area abutting the midline. (5l) CycA RNA expression in robo embryos. The cells expressing cyclins are located as in WT.

The cell cyclin clusters show changes in transcript levels of several cyclins (CycA, CycB, cdc2, CycB3, fzy) in both mutant and transgenic embryos. Most notably, the microarray data indicates that for all the robo GOF, robo overexpression is always accompanied by an increase in the transcription of cyclins. Hence we decided to verify if indeed cyclin RNA and protein are increased in robo GOF embryos. We assessed the precise location of these expression changes through in situ hybridization. To verify that changes in cyclin transcript levels correlate with changes in protein levels, we employed immunocytochemistry with various antibodies against cell cyclins. We analyzed the RNA and protein patterns for cyclins (Figure 5) in three robo GOF: (a) UR.mef – robo GOF is overexpressed in all muscles (mef2 driver), (b) UR.yf – a stronger robo than wild-type (robo-yf) is overexpressed in the CNS (elav driver), and (c) FRM – the cytoplasmic domain of robo is overexpressed in the CNS (elav driver). In agreement with the microarray data, robo overexpression in post-mitotic neurons (driver elav) is accompanied with a large increase in cyclin expression both at the level of RNA and protein (black arrows in Figure 5c,5d, and 5e). The increase in cyclin expression is especially clear when overexpressing robo in the muscles (driver mef2), making it visible in the pleural external oblique muscles (5f) and the dorsal external muscles (5g). These findings support the hypothesized link between cell cyclins and axon guidance.

The microarray data does not indicate a significant change in cell cyclin expression in axon guidance mutants. But in view of the robo GOF results, we decided to analyze the cyclin expression in the axon guidance mutants slit, robo, and robo12. Consistent with the microarray data, there are no visible quantitative cyclin expression changes in these mutants. Yet several major anatomical rearrangements in the cyclin expressing cells are evident. In wild type embryos, cyclin staining is visible from the dorsal side up to and at the midline. In the mutants for slit (5j) and robo12 (5k), there is a distinct gap in cyclin staining around the midline, and no dorsal cyclin-expressing cells are visible. At the midline, slit and robo12 mutants show WT-like cyclin staining. In robo mutants (5l) no apparent change is observed.

## Conclusion

Midline crossing in Drosophila is a model of choice for axon guidance, and yet we are only beginning to understand the role of transcription in the control of axon guidance. Control does occur through protein translation. In such a spatially and temporally fine-tuned process, it is intuitive that transcription would also be involved. Previous work in flies and mice points in this direction; the role of transcription factors is also emerging as very relevant, as reviewed in [27]. To investigate this topic we have undertaken a whole-genome expression study of mutants and transgenics of the key axon guidance genes. If control of midline crossing proceeds through transcription, the genetic perturbations induced in the mutants and transgenics should reveal clusters enriched for genes known to be involved in axon guidance. We also investigated the expression patterns of known axon guidance genes during normal development in a wild type microarray time course, using a previously published microarray data set.

We have found that the *hopach *package provides a robust, yet flexible, approach to clustering gene expression data. *hopach *is one of several hybrid algorithms [28] which are gaining popularity in the microarray community. The extension of *hopach *proposed here has the advantage that all genes can be assigned to a cluster, even when all pair-wise distances cannot be stored in computer memory. Also, the assignment of genes to clusters are based on bootstrap membership values, accounting directly for variability and making results reproducible [11].

To characterize clusters, we recommend integrating gene annotations from multiple complementary controlled vocabularies. In this study we used GO and APoGE, which combine the bulk of literature knowledge with nascent efforts towards a complete genetic expression characterization in Drosophila. When co-expression in microarray data is coupled with a shared RNA expression pattern and/or shared terms in the GO vocabulary, gene annotation via the "guilt by association" paradigm utilized so often in functional genomics becomes a much less tenuous method. For a specific cluster space, we used two independent data sets – a mutant/transgenic data set and a time course data set – and focused on genes which are co-expressed on both data sets. This approach is conceptually similar to techniques where two different axes are used, such as 2-D electrophoresis protein separation.

Our analysis revealed clear co-expression of axon guidance genes in both the GOF/LOF data and the developmental time course. This finding indicates that transcription does in fact play a role in control of midline crossing. We identified several clusters containing many genes known to be relevant in midline crossing, but also numerous less well characterized genes. Cluster AG132 is one of the most interesting clusters; genes such as dock, plexB, Cam, CadN, caps, spen, Fs(2)Ket, Con, RhoGAP19D, Rac2, and shot belong to this cluster. By querying APoGE for spatial/temporal expression patterns of interest, we were able to immediately identify several dozen candidates for further studies. We have selected a pool of candidates meeting both of the following criteria: 1) membership in a selected axon guidance cluster (as discussed in this paper), and 2) a known pattern of expression in the wild-type CNS, according to APoGE. In short, these genes are present in the right time and place to be involved in axon guidance, and further, their expression patterns are perturbed when the guidance process is disrupted. This candidate pool is thus a very attractive target for genetic analysis, the more so because many candidate genes are already represented by mutant stocks in publically available collections. Sensitized genetic backgrounds, such as the slit and robo heterozygotes, have been successfully used in the past to identify additional genes involved in pathfinding [29]. We suggest using trans-heterozygote analysis to search for interactions between (1) candidate genes within the same cluster (such as AG132, AG164, and TC91), and (2) candidate genes and key axon guidance genes (slit, robo, etc.)

Our analysis suggests that known axon guidance genes may also be involved in hemocyte migration. Cluster AG164 contains a significant number of genes expressed in hemocytes, the Drosophila blood cells. Hemocytes migrate during development, with the VEGF pathway involved in the guidance process. We observed through RNA expression analysis that hemocyte migration does not proceed normally in slit and slit.robo – hemocytes are absent in the central segments of the embryo. In robo12, a double mutant for genes robo and robo2 (the known receptors for slit), hemocyte migration is normal, even though the CNS phenotype is the same as in slit and slit.robo. Hence, we speculate that slit may function independently of its robo receptors in this context. Blood vessel migration has been linked in mammals to the same molecular processes as axon guidance during recent studies [30]. Both slit and robo have been implicated in several ways in the vascularization system of vertebrates [31,32]. Since vascularization is a more recent evolutionary development than axon guidance [33,30], it appears that some of the molecules involved in axon guidance may have been co-opted by vascularization mechanisms fairly early during evolution. Prompted by the observation that numerous cell cyclin genes are present in a few clusters in our microarray data, we analyzed RNA and protein patterns of cyclins in axon guidance mutants and transgenics. We examined several robo GOF (UR.mef, UR.yf, FRM), because they show the strongest overexpression in the cyclin cluster of the axon guidance microarray data. We also studied the LOF mutants of each of the key axon guidance genes slit, robo and robo12. The overexpression of robo is accompanied with an increase in RNA and protein levels of several cyclins. We also observe that cyclin expressing cells are dislocated in slit and in the double robo12 mutants. The cyclin expressing cells are visible away from the midline, exactly the opposite direction of the axons that are stalled at the midline in these two mutants. Our results suggest that coordinated expression of cyclins may play a role in timing the midline crossing process. The role of cell cyclins might be to adequately time the developmental state of the neurons and to assure that all the multiple signaling pathways for axon guidance are working in the proper time and location, similar to the role cyclins play in the checkpoints in cell division [23].

In conclusion, this study has shown that axon guidance is under transcriptional control. We have also observed that genes transcriptionally regulated in axon guidance mutants and transgenics are expressed in hemocytes, indicating that blood vessel migration may employ a mechanism involving axon guidance molecules. Furthermore, our results suggest that coordinated expression of cell cyclins may play a role in timing the midline crossing process. Together, these results provide new insights into the roles of axon guidance genes in vivo and demonstrate the validity of our whole genome approach as a method to study transcriptional networks in other biological systems.

## Methods

### Drosophila Stocks

We used 17 mutants and transgenic animals in this experiment. Flies were placed in cages at room temperature and plates were changed every hour. We collected embryos at late stage 15, and visually assessed before RNA extraction. For lethal mutations, the CyO-Kruppel GFP balancer was used [34] and the homozygous mutants were selected against fluorescence under a UV microscope. All mutants are available from Bloomington.

WT flies are Canton-S flies. comm embryos result from crossing Comm^Δe39^[35] with comm^P ^[36]. comm^Δe39 ^is a null allele. comm^P ^is a 900 bp deletion of the 5' UTR and the transcription start site. comm/WT was obtained by crossing comm^Δe39 ^with WT flies. The robo LOF is robo^4 ^and is a point mutation [35]. robo.WT was obtained by crossing robo^4^with WT flies. robo2^x123 ^results from an excision of EP 2582 [4]. robo12 is the robo, robo2 double mutant resulting from the recombination of robo^4 ^with robo2^x123 ^[4]. slit^2 ^comes from an EMS screen and is a point mutation [37]. slit.WT results from crossing slit^2 ^with WT flies. slit.robo was obtained by recombination of slit^1 ^with robo^4^[29]. UC is the comm GOF, and corresponds to a single copy of the UAS-comm construct crossed with the driver elavGal4 [3]. UC2x is a stronger comm GOF and was obtained using two copies of UAS-comm crossed with the driver elavGal4 (this work). UR is a robo GOF that results from crossing UAS-robo flies with the postmitotic neural driver elavGal4 flies [38]. UR.yf is a phenotypically stronger robo GOF obtained from the cross of UAS-robo-yf flies with the postmitotic driver elavGal4 [39]. UR.mef designates embryos with robo overexpression in the muscles, produced by crossing UAS-robo-yf flies with the mesodermal driver mef2Gal4 [40]. UR2 is a robo2 GOF obtained by crossing the construct UAS-robo2 with the elavGal4 driver [4]. FRM is a robo GOF, obtained by crossing the driver elavGal4 with a UAS chimeric construct, which combines the robo cytoplasmic domain with the frazzled extracellular domain [41]. UNC5 is the unc-5 GOF, obtained by crossing, a GS-element insert located upstream of unc-5, with the elavGal4 (GSunc5 kindly provided by John Thomas).

### Developmental Time Course Stocks

The time course data set was published and details reported in [10]. There are 12 samples of 3 replicates each of non-overlapping 1 hour collection, starting from 30 to 90 minutes and ending at 11.5 to 12.5 hours post egg laying.

### DNA Microarrays and Target Preparation

RNA was extracted from the embryos using QIAGEN columns according to manufacturer recommendations. Embryonic RNA was hybridized to Affymetrix DrosGenome1 microarrays according to the standard Affymetrix protocols.

### Software

All analysis was performed in the R statistical programming language (v2.0.0), available at [42], with the Bioconductor (release 1.5) add-on packages *rma*, *siggenes*, and *hopach*, available at [43].

### Data Processing

Expression measures were calculated using the RMA algorithm in the package *rma*, applied as indicated in [44], with the recommended quantile-quantile normalization procedure. The *siggenes *package was used to rank all genes based on differential expression (log2 ratio) relative to a control, which was RNA from WT flies. The top ranked most differentially expressed genes in each condition (mutant or transgenic) were selected until their number totaled 2000 for each experiment. These provided the initial seed data set for hierarchical clustering.

### Clustering with hopach

*hopach *is a hybrid hierarchical clustering method specifically designed for analysis of microarray data. The algorithm builds a non-binary, hierarchical tree, but also assigns genes to clusters [11]. The final level of the tree provides a meaningful ordered list in which nearby genes are similarly expressed. The first level of the tree with maximal average cluster homogeneity is identified and cluster labels are assigned based on the partitioning of genes in this level. Then, each gene's membership in every cluster is assessed using a non-parametric bootstrap method that fixes the cluster profiles, and for each of many resampled data sets reassigns every gene to the cluster whose profile is closest. The proportion in which a gene appears in each cluster is a measure of its cluster membership. Thus, the variability of the estimated cluster labels can be directly assessed from a single data set through this resampling approach.

We adapted the *hopach *methodology as follows:

#### 1. Initial clusters

Apply the standard *hopach *algorithm with an appropriate choice of distance to a set of several thousand pre-selected genes. This produces initial clusters, each represented by a medoid gene.

#### 2. Bootstrap extended clusters

Run the non-parametric bootstrap with the fixed medoids (from the previous step) as described in [11] to obtain a percentage membership value (between 0 and 1, with 1 meaning that a gene only belongs to that cluster) for each gene in every cluster. Reassign genes to clusters based on an appropriate threshold for bootstrap membership. Possible values include > 0.8 (genes belong to only one cluster, very homogenous clusters), > 0.5 (genes belong to only one cluster, less homogeneous) and > 0.3 (genes can be present in more than one cluster – fuzzy clustering). Note that this step is performed using *all *genes in the data set (not just selected genes), so that every gene is now assigned to a cluster.

#### 3. Final clusters

Optionally, apply *hopach *again to every extended cluster to produce a new set of sub-clusters. In general, large clusters will be further divided at this step, whereas smaller ones will not. When the membership threshold in Step 2 is large enough that genes belong to only one cluster, this second application of *hopach *can be used to create a full hierarchical tree and a unique, final ordering of all genes. If this step is skipped, the bootstrap extended clusters are the final clusters.

#### 4. Order within clusters

Reorder the genes in each cluster based on distance to the medoid – the medoid for each cluster is thus ranked one, genes with low ranks form the core of the cluster, and genes with high ranks are more peripheral.

This modified *hopach *method was applied to each data set separately, beginning with 2000 selected genes and including all genes on the Affymetrix DrosGenome1 microarray in Step 2. We used cosine-angle (uncentered correlation) distance (Step 1) and a membership threshold of 0.3 (Step 2).

### Controlled Vocabularies

The vocabularies for GO were downloaded from [45]. APoGE vocabulary can be obtained upon request. We selected terms relevant to axon guidance: "axon guidance" (GO:0007411) in GO; "ventral nerve cord", "embryonic brain" and "ventral midline" in APoGE. Over representation in each cluster was evaluated using the hypergeometric distribution. Clusters of size 10 or larger were ordered based on hypergeometric p-value, which gives the probability of finding by chance alone the observed number of genes or more in that cluster annotated with the term (conditional on the total number of genes, the size of the cluster, and the frequency of the annotation over all genes). For each term, this produces one p-value for each cluster. For the most interesting clusters, we queried all the available pictures in APoGE, searching for patterns consistent with relevance to axon guidance mechanisms.

### In Situ Hybridization and Antibody Staining

In situ hybridization were done to assess RNA expression of several specific genes. The experiment was conducted in 96 well plates and followed the protocol published in [10]. Antibody staining of whole mount embryos followed the standard procedure outlined in [46,29]. BP102 (a gift from Corey Goodman) is a specific antibody for the Drosophila embryo central nervous system. B102 was used at concentrations of 1:10. The antibodies against the cyclins CycA, CycB, CycB3 and Cdc2 were kindly provided by Patrick O'Farrel and were used at concentrations of 1:5, 1:5, 1:5 and 1:4. The HRP-conjugated secondary antibodies [Jackson Labs] were used at a 1:250 dilution.

## Authors' contributions

TRM designed the experiment, carried out the microarray hybridizations and analysis, and performed the in situ and protein hybridizations. JP participated in experiment design and in the in situ and protein hybridizations. PT participated in the design of the study and is the responsible for the APoGE database. KSP contributed to design of the study, microarray analysis and writing the manuscript. All authors read and approved the final manuscript.

## Supplementary Material

Additional file 1 Axon Guidance Cluster 132. Annotations and In situ images for genes in Cluster AG132. Click here for file

Additional file 2 Axon Guidance Cluster 164. Annotations and In situ images for genes in Cluster AG164. Click here for file

Additional file 3 Time Course Cluster 91. Annotations and In situ images for genes in Cluster TC91. Click here for file

Additional file 4 Hopach Clustering Results. Tables of gene cluster assignments for axon guidance and time course microarray data sets. Click here for file
