# Supplementary material for: Transcriptional control in embryonic Drosophila midline guidance assessed through a whole genome approach
Source: BMC Neurosci. 2007 Jul 31;8:59. doi: 10.1186/1471-2202-8-59 (PMC1950096; doi:10.1186/1471-2202-8-59)
Supplement: Additional file 1 — Axon Guidance Cluster 132. Annotations and In situ images for genes in Cluster AG132. [file 1471-2202-8-59-S1.pdf]

Table 1: Relevant Genes in Cluster AG132

Symbol	Notes	BRP	Interactions	Reference
InR	axon guidance; insulin receptor; regulation of cell growth; phe in commissures	0.507	dock; chico	[32]
plexB	axon guidance; Rac, Rho signaling	0.745	Pak, Rac1, Rho1, trio	[13]
spen	axon guidance; glia cell migration; phe affect longitudinals; expr in commissures	0.561	aop	[17]
CadN	axon guidance; cell-cell adhesion; expr in commissures; phe in longitudinals	0.603		[19]
dock	axon guidance; insulin receptor; expr in growth cone; phe in axon guidance	0.346	Dscam; InR; trio; Pak	[11] [7]
Cam	axon guidance; expr in CNS; mutations show defects in sense organs	0.888		[35]
caps	axon guidance; cell adhesion; expr in neurons; phe in SNb pathfinding	0.431	kr	[1]
gish	glia cell migration; phe in glia migrations and axon pathfinding	1.000		[14]
Src64B	CNS dev; MB dev; phe axon growth and guidance	0.908		[22]
Con	cell-cell adhesion; axonal fasciculation; SNb pathfinding; expr in commissures	0.774	Fas2, sema1-a	[23]
glec	cell-cell adhesion; expr in midline; phe affect commissures; axon pathfinding	0.499		[30]
Rac2	GTPase activity; cytoskeleton; 2% show midline guidance defects	0.444	trio, robo, Rac1	[12]
Fs(2)Ket	actin organization; phe in axon guidance	0.909		[18]

Continued on next page

Symbol	Notes	BRP	Interactions	Reference
shot	cytoskeleton; axon extension; expr in commissures; phe in longitudinals	0.867		[20]
elav	neurogenesis; expr in CNS; maintenance of neurons; phe in commissures	0.689		[15]
pum	synaptic transmission; cell migration; phe in photoreceptor targeting	0.828		[29]
G-oα47A	GTPase; phe in longitudinals; pathways of motorneurons	0.740		[10]
mask	cytoskeleton; photoreceptor differentiation; phe R7	0.926		[31]
Sap47	synaptic transmission	0.952		[27]
SP1070	cell-cell adhesion; Notch binding; proliferation; neurogenesis	0.786		
cpo	PNS development; expr in CNS	0.554		[3]
LIMK1	actin cytoskeletal reorganization; neurogenesis	0.403	RhoGEF2, cofilin	[24]
Gbeta13F	GTPase; actin organization; phe in neuroblasts; expr in CNS	0.676		[28]
Dl	cell-cell adhesion; neurogenesis; CNS and PNS development	0.361	Abl	[6]
CG3654	neurogenesis	0.701		
stich1	PNS development	0.734		
milton	axon transport of mitochondria	0.453		[33]
bun	PNS development	0.673		
l(2)gl	neurogenesis; establishment of neuroblast; cytoskeleton	0.390	Abl, Fas1, sema-5C	[9]
h	neurogenesis	0.553		
RhoGAP19D	small GTPase	0.704		[5]
CG11727	GTPase	0.481		[4]

Continued on next page

Symbol	Notes	BRP	Interactions	Reference
Dlic2	cytoskeleton	0.870		
Glt	basement membrane; expr in CNS	0.501		[25]
Lk6	microtubule; kinase	0.362		[16]
Toll-6	cell-cell adhesion; defense response	0.385		
R	cell-cell adhesion; GTPase activity	0.788		
rin	eye development; ras signaling	0.846		[26]

Note: phe – phenotype; expr – expressed; MB – Mushroom Bodies; dev – development; NMJ – Neuromuscular Junction; tf – transcription factor

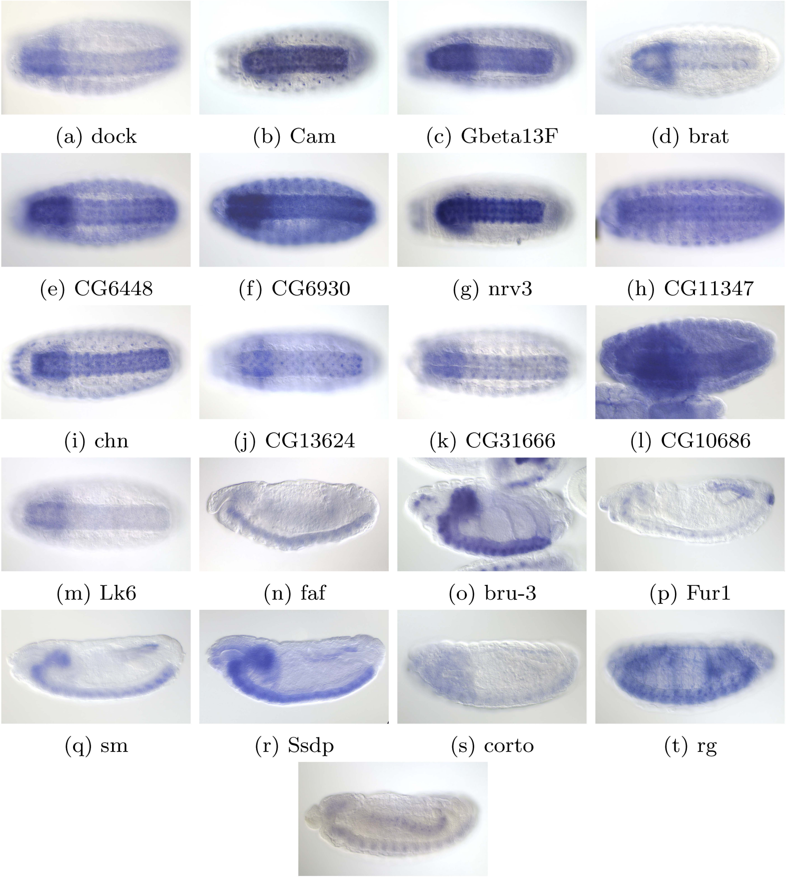

(a) dock (b) Cam (c) Gbeta13F (d) brat

(e) CG6448 (f) CG6930 (g) nrv3 (h) CG11347

(i) chn (j) CG13624 (k) CG31666 (l) CG10686

(m) Lk6 (n) faf (o) bru-3 (p) Fur1

(q) sm (r) Ssdp (s) corto (t) rg

(u) G-oalpha47A

Figure 1: **APoGE In Situ Hybridization for Cluster AG132**

Figure 1: **APoGE In Situ Hybridization for Cluster AG132 (cont.)** APoGE was queried for genes in Cluster AG132. There are APoGE entries for 77 of the 161 genes of Cluster AG132; (1a) to (1m) (13 genes) show similar expression in the embryonic brain and the ventral nerve cord. (1n) to (1u) are 8 genes mentioned as expressed in the same two regions, but whose available pictures are only lateral views. (1a) to (1m) are shown ventrally. (1n) to (1u) are shown laterally. (1a) dock is an adaptor protein, with an important key role in axon guidance and whose mutants show severe axon guidance defects [11]. (1b) Cam has been implicated in photoreceptor light termination, muscle synapses and midline crossing [35]. (1c) Gbeta13F mutants have neuroblasts defects [28]. (1d) Brat is involved in the regulation of cellular rRNA and brat mutants show brain tumors [2]. (1e) CG6448 and (1f) CG6930 have has no information available. (1g) nrv3 is a sodium:potassium-exchanging ATPase. (1h) CG11347 has no information available. (1i) chn is putatively involved in neurogenesis. (1j) CG13624, (1k) CG31666, and (1l) CG10686 are genes for which no information is available. (1m) Lk6 is involved in microtubule function [16]. (1n) faf is involved in photoreceptor fate and synapse regulation [8]. (1o) bru-3 is involved in RNA binding. (1p) Fur1 is relevant in proteolysis and peptidolysis. (1q) sm is a gene involved in mRNA processing. (1r) Ssdp is a DNA binding protein, that interacts with LIM proteins [34]. (1s) corto is a hox genes regulator, whose mutations affect the adult brain [21]. (1t) rg is involved in cone cell differentiation. (1u) G-oα47A is a GTPase, important in heart development; mutants have a motor axon guidance defect [10].

References

[1] S. Abrell and H. Jackle. Axon guidance of drosophila snb motoneurons depends on the cooperative action of muscular kruppel and neuronal capricious activities. *Mech Dev*, 109(1):3–12, 2001.

[2] E. Arama, D. Dickman, Z. Kimchie, A. Shearn, and Z. Lev. Mutations in the beta-propeller domain of the drosophila brain tumor (brat) protein induce neoplasm in the larval brain. *Oncogene*, 19(33):3706–16, 2000.

[3] H. J. Bellen, S. Kooyer, D. D'Evelyn, and J. Pearlman. The drosophila couch potato protein is expressed in nuclei of peripheral neuronal precursors and shows homology to rna-binding proteins. *Genes Dev*, 6(11):2125–36, 1992.

[4] A. Bernards. Gaps galore! a survey of putative ras superfamily gtpase activating proteins in man and drosophila. *Biochim Biophys Acta*, 1603(2):47–82, 2003.

[5] P. Billuart, C. G. Winter, A. Maresh, X. Zhao, and L. Luo. Regulating axon branch stability: the role of p190 rhogap in repressing a retraction signaling pathway. *Cell*, 107(2):195–207, 2001.

[6] D. Crowner, M. Le Gall, M. A. Gates, and E. Giniger. Notch steers drosophila isnb motor axons by regulating the abl signaling pathway. *Curr Biol*, 13(11):967–72, 2003.

[7] C. J. Desai, P. A. Garrity, H. Keshishian, S. L. Zipursky, and K. Zinn. The drosophila sh2-sh3 adapter protein dock is expressed in embryonic axons and facilitates synapse formation by the rp3 motoneuron. *Development*, 126(7):1527–35, 1999.

[8] A. DiAntonio, A. P. Haghighi, S. L. Portman, J. D. Lee, A. M. Amaranto, and C. S. Goodman. Ubiquitination-dependent mechanisms regulate synaptic growth and function. *Nature*, 412(6845):449–52, 2001.

[9] T. Elkins, K. Zinn, L. McAllister, F. M. Hoffmann, and C. S. Goodman. Genetic analysis of a drosophila neural cell adhesion molecule: interaction of fasciclin i and abelson tyrosine kinase mutations. *Cell*, 60(4):565–75, 1990.

[10] F. Fremion, M. Astier, S. Zaffran, A. Guillen, V. Homburger, and M. Semeriva. The heterotrimeric protein go is required for the formation of heart epithelium in drosophila. *J Cell Biol*, 145(5):1063–76, 1999.

[11] P. A. Garrity, Y. Rao, I. Salecker, J. McGlade, T. Pawson, and S. L. Zipursky. Drosophila photoreceptor axon guidance and targeting requires the dreadlocks sh2/sh3 adapter protein. *Cell*, 85(5):639–50, 1996.

[12] S. Hakeda-Suzuki, J. Ng, J. Tzu, G. Dietzl, Y. Sun, M. Harms, T. Nardine, L. Luo, and B. J. Dickson. Rac function and regulation during drosophila development. *Nature*, 416(6879):438–42, 2002.

[13] H. Hu, T. F. Marton, and C. S. Goodman. Plexin b mediates axon guidance in drosophila by simultaneously inhibiting active rac and enhancing rhoa signaling. *Neuron*, 32(1):39–51, 2001.

[14] T. Hummel, S. Attix, D. Gunning, and S. L. Zipursky. Temporal control of glial cell migration in the drosophila eye requires gilgamesh, hedgehog, and eye specification genes. *Neuron*, 33(2):193–203, 2002.

[15] F. Jimenez and J. A. Campos-Ortega. Genes in subdivision 1b of the drosophila melanogaster x-chromosome and their influence on neural development. *J Neurogenet*, 4(4):179–200, 1987.

[16] D. Kidd and J. W. Raff. Lk6, a short lived protein kinase in drosophila that can associate with microtubules and centrosomes. *J Cell Sci*, 110 (Pt 2):209–19, 1997.

[17] B. Kuang, S. C. Wu, Y. Shin, L. Luo, and P. Kolodziej. split ends encodes large nuclear proteins that regulate neuronal cell fate and axon extension in the drosophila embryo. *Development*, 127(7):1517–29, 2000.

[18] J. P. Kumar, G. S. Wilkie, H. Tekotte, K. Moses, and I. Davis. Perturbing nuclear transport in drosophila eye imaginal discs causes specific cell adhesion and axon guidance defects. *Dev Biol*, 240(2):315–25, 2001.

[19] C. H. Lee, T. Herman, T. R. Clandinin, R. Lee, and S. L. Zipursky. N-cadherin regulates target specificity in the drosophila visual system. *Neuron*, 30(2):437–50, 2001.

[20] S. Lee, K. L. Harris, P. M. Whitington, and P. A. Kolodziej. short stop is allelic to kakapo, and encodes rod-like cytoskeletal-associated proteins required for axon extension. *J Neurosci*, 20(3):1096–108, 2000.

[21] A. Lopez, D. Higuet, R. Rosset, J. Deutsch, and F. Peronnet. corto genetically interacts with pc-g and trx-g genes and maintains the anterior boundary of ultrabithorax expression in drosophila larvae. *Mol Genet Genomics*, 266(4):572–83, 2001.

[22] M. Nicolai, C. Lasbleiz, and J. M. Dura. Gain-of-function screen identifies a role of the src64 oncogene in drosophila mushroom body development. *J Neurobiol*, 57(3):291–302, 2003.

[23] A. Nose, T. Umeda, and M. Takeichi. Neuromuscular target recognition by a homophilic interaction of connectin cell adhesion molecules in drosophila. *Development*, 124(8):1433–41, 1997.

[24] K. Ohashi, T. Hosoya, K. Takahashi, H. Hing, and K. Mizuno. A drosophila homolog of lim-kinase phosphorylates cofilin and induces actin cytoskeletal reorganization. *Biochem Biophys Res Commun*, 276(3):1178–85, 2000.

[25] P. F. Olson, L. I. Fessler, R. E. Nelson, R. E. Sterne, A. G. Campbell, and J. H. Fessler. Glutactin, a novel drosophila basement membrane-related glycoprotein with sequence similarity to serine esterases. *Embo J*, 9(4):1219–27, 1990.

[26] C. Pazman, C. A. Mayes, M. Fanto, S. R. Haynes, and M. Mlodzik. Rasputin, the drosophila homologue of the rasgap sh3 binding protein, functions in ras- and rho-mediated signaling. *Development*, 127(8):1715–25, 2000.

[27] C. Reichmuth, S. Becker, M. Benz, K. Debel, D. Reisch, G. Heimbeck, A. Hofbauer, B. Klagges, G. O. Pflugfelder, and E. Buchner. The sap47 gene of drosophila melanogaster codes for a novel conserved neuronal protein associated with synaptic terminals. *Brain Res Mol Brain Res*, 32(1):45–54, 1995.

[28] M. Schaefer, M. Petronczki, D. Dorner, M. Forte, and J. A. Knoblich. Heterotrimeric g proteins direct two modes of asymmetric cell division in the drosophila nervous system. *Cell*, 107(2):183–94, 2001.

[29] D. Schmucker, H. Jackle, and U. Gaul. Genetic analysis of the larval optic nerve projection in drosophila. *Development*, 124(5):937–48, 1997.

[30] M. Sharrow and M. Tiemeyer. Gliolectin-mediated carbohydrate binding at the drosophila midline ensures the fidelity of axon pathfinding. *Development*, 128(22):4585–95, 2001.

[31] R. K. Smith, P. M. Carroll, J. D. Allard, and M. A. Simon. Mask, a large ankyrin repeat and kh domain-containing protein involved in drosophila receptor tyrosine kinase signaling. *Development*, 129(1):71–82, 2002.

[32] J. Song, L. Wu, Z. Chen, R. A. Kohanski, and L. Pick. Axons guided by insulin receptor in drosophila visual system. *Science*, 300(5618):502–5, 2003.

[33] R. S. Stowers, L. J. Megeath, J. Gorska-Andrzejak, I. A. Meinertzhagen, and T. L. Schwarz. Axonal transport of mitochondria to synapses depends on milton, a novel drosophila protein. *Neuron*, 36(6):1063–77, 2002.

[34] D. J. van Meyel, J. B. Thomas, and A. D. Agulnick. Ssdp proteins bind to lim-interacting co-factors and regulate the activity of lim-homeodomain protein complexes in vivo. *Development*, 130(9):1915–25, 2003.

[35] M. F. VanBerkum and C. S. Goodman. Targeted disruption of ca(2+)-calmodulin signaling in drosophila growth cones leads to stalls in axon extension and errors in axon guidance. *Neuron*, 14(1):43–56, 1995.
