# Supplementary material for: Transcriptional control in embryonic Drosophila midline guidance assessed through a whole genome approach
Source: BMC Neurosci. 2007 Jul 31;8:59. doi: 10.1186/1471-2202-8-59 (PMC1950096; doi:10.1186/1471-2202-8-59)
Supplement: Additional file 2 — Axon Guidance Cluster 164. Annotations and In situ images for genes in Cluster AG164. [file 1471-2202-8-59-S2.pdf]

Table 1: Relevant Genes in Cluster AG164

Symbol	Notes	BRP	Interactions	Reference
trio	axon guidance; CNS; Rho transduction; phe axon pathways	0.939	Abl; Rac1; Dock	[23] [1]
robo3	axon guidance; cell-cell adhesion; VNC development; phe in connectives	0.344	Abl	[26]
Nedd4	axon guidance; phe in commissures	0.701	comm	[22]
Egfr	axon guidance; brain dev; phe in axon guidance; axonal connectivity	0.477	neuroglian; vvl	[9] [10]
lola	axon guidance; phe in longitudinals; tf	0.447		[8]
side	axon guidance; cell adhesion	0.652		[29]
Fmr1	axon guidance; synaptogenesis; translation	0.568		[21]
MICAL	axon guidance; actin binding; phe in fascicles	0.405		[33]
WASp	axon guidance; axonal fasciculation; cytoskeleton; actin binding	0.746		[32]
shg	cell-cell adhesion; neurogenesis	0.787		[35]
vvl	PNS dev; axonal connectivity; expr CNS; migration of midline glia; phe in longitudinals	0.662	Egfr	[16]
CG4383	cell-cell adhesion; axon guidance by similarity with semaphorin 2a	0.698		
stg	PNS dev; phe in commissures; neurogenesis	0.997		[34] [20]
shi	cytoskeleton; GTPase; expr in CNS; vesicular transport; phe in commissures	0.575	slit	[24]
ed	cell-cell adhesion; neurogenesis; phe in VNC	0.752	Egfr; aop; ttk	
raw	PNS dev; expr in CNS; phe in CNS	0.526		[11]

Continued on next page

Symbol	Notes	BRP	Interactions	Reference
SoxN	neurogenesis; tf; phe in commissures	0.479		[5]
pbl	cell adhesion; PNS dev; Rho exchange; phe in anterior fascicle	0.345	RacGAP50C; Rho1	[31] [25]
babo	CNS dev; phe MB; synaptic growth	0.570	EcR Smox	[19]
Liprin-α	synapse; cell adhesion; phe in R7 projection	0.809		[13]
salm	expr in CNS; phe in CNS	0.692		[7]
RhoGAP88C	GTPase activator; actin filament organization	0.825		[3]
Nf1	Ras GTPase; learning and memory; phe in short term memory and NMJ	0.658		[36]
cenG1A	GTPase	0.348		
RhoGAP18B	GTPase; actin organization	0.599		[3]
CG32560	RasGTPase	0.758		
RhoGAPp190	RhoGTPase activator; MB dev	0.559		[3]
CG31048	small GTPase	0.394		
pk	cytoskeleton; expr ventral midline; drive omatidium	0.897	Rac1	[40]
CG6735	cytoskeleton	0.749		[14]
CG32138	cytoskeleton; intracellular signaling cascade	0.453		
cher	cytoskeleton	0.773		
Syn2	cytoskeleton	0.551		
spir	cytoskeleton; Rho signaling with actin	0.852	Rac1, Cdc42	
CG5169	actin filament	0.412		[15]
mask	cytoskeleton; photoreceptor differentiation	0.631		[30]
robl	MB dev; cytoskeleton	0.965		[27]
Fim	cytoskeleton; actin binding	0.737		

Continued on next page

Symbol	Notes	BRP	Interactions	Reference
bon	PNS dev; tf	0.521		[28]
Arp66B	cytoskeleton; axonal fasciculation; phe in commissures	0.526		[39]
SP2353	cell-cell adhesion	0.590		
CG7166	cell-cell adhesion	0.643		
CG16974	cell adhesion	0.720		
crol	cell adhesion; tf; regulates integrin; phe fascicle	0.416	ecdysione, EcR	
EcR	neuronal remodeling; tf; cell adhesion; phe MB	0.425	ecdysione, crol, babo	[17]
CG18249	cell adhesion	0.349		
mys	cell adhesion; CNS dev	0.622		
klg	R7 differentiation; cell adhesion; expr in CNS	0.469		[6]
CrebB-17A	long-term memory; synaptic plasticity	0.366	Fas2	[38]
Eps-15	vesicle transport	0.726		
Klp64D	cytoskeleton; synaptic transport; kinesin	0.553		
CG7343	mutations affect the eye; synaptic transport; growth	0.845		[12]
syd	axon cargo transport	0.616		[4]
zetaCOP	vesicle-mediated transport	0.601		

Note: phe – phenotype; expr – expressed; MB – Mushroom Bodies; dev – development; NMJ – Neuromuscular Junction; tf – transcription factor

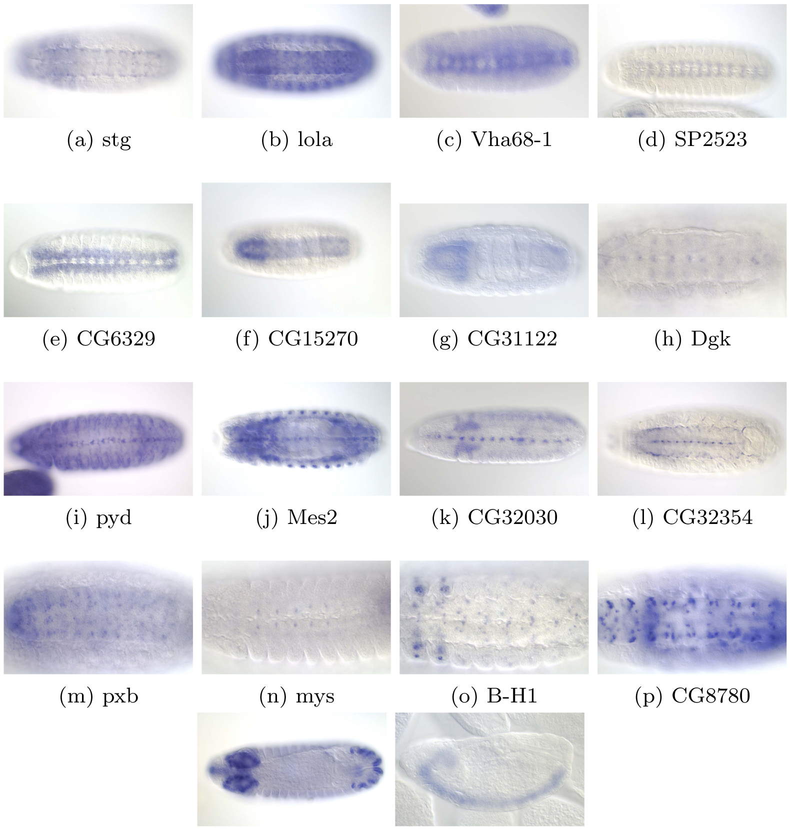

(a) stg (b) lola (c) Vha68-1 (d) SP2523

(e) CG6329 (f) CG15270 (g) CG31122 (h) Dgk

(i) pyd (j) Mes2 (k) CG32030 (l) CG32354

(m) pxb (n) mys (o) B-H1 (p) CG8780

(q) klu (r) CG2225

Figure 1: **APoGE In Situ Hybridization for Cluster AG164 (cont.)** APoGE was queried for genes belonging to Cluster AG164. There are APoGE entries for 140 of the 281 genes belonging to Cluster AG164. (1a) to (1p) are shown ventrally. (1q) shown dorsally. (1r) is shown laterally. Genes depicted in (1a) to (1g) show a distinct VNC expression with longitudinal and commissural staining. Genes depicted in (1h) to (1m) show midline staining. Genes in images (1n) to (1r) have VNC and embryonic brain expression. (1a) stg is a cell cyclin gene which has a commissure phenotype [20]. (1b) lola is an axon guidance gene, with reported longitudinal defects [18]. (1c) Vha68-1 has hydrogen-exporting ATPase activity. (1d) No useful information exists for SP2523, (1e) CG6329, (1f) CG15270, and (1g) CG31122. (1h) Dgk is a diacylglycerol kinase. (1i) pyd is a gene with guanylate kinase activity, involved in cell fate choices. (1j) No information is available on Mes2. (1k) CG32030 shows homology to actin binding genes. (1l) No information exists for CG32354. (1m) pxb is part of the smoothened signaling pathway. (1n) mys is an integrin implied in CNS development [2]. (1o) B-H1 is a homeobox gene involved in eye development. (1p) No useful information exists on CG8780. (1q) klu is involved in neuroblast differentiation [37]. (1r) There is no information for CG2225.

References

[1] J. Bateman, H. Shu, and D. Van Vactor. The guanine nucleotide exchange factor trio mediates axonal development in the drosophila embryo. *Neuron*, 26(1):93–106.

[2] K. J. Beumer, J. Rohrbough, A. Prokop, and K. Broadie. A role for ps integrins in morphological growth and synaptic function at the postembryonic neuromuscular junction of drosophila. *Development*, 126(24):5833–46.

[3] P. Billuart, C. G. Winter, A. Maresh, X. Zhao, and L. Luo. Regulating axon branch stability: the role of p190 rhogap in repressing a retraction signaling pathway. *Cell*, 107(2):195–207.

[4] A. B. Bowman, A. Kamal, B. W. Ritchings, A. V. Philp, M. McGrail, J. G. Gindhart, and L. S. Goldstein. Kinesin-dependent axonal transport is mediated by the sunday driver (syd) protein. *Cell*, 103(4):583–94.

[5] M. Buescher, F. S. Hing, and W. Chia. Formation of neuroblasts in the embryonic central nervous system of drosophila melanogaster is controlled by soxneuro. *Development*, 129(18):4193–203.

[6] S. J. Butler, S. Ray, and Y. Hiromi. klingon, a novel member of the drosophila immunoglobulin superfamily, is required for the development of the r7 photoreceptor neuron. *Development*, 124(4):781–92.

[7] R. Cantera, K. Luer, T. E. Rusten, R. Barrio, F. C. Kafatos, and G. M. Technau. Mutations in spalt cause a severe but reversible neurodegenerative phenotype in the embryonic central nervous system of drosophila melanogaster. *Development*, 129(24):5577–86.

[8] D. Crowner, K. Madden, S. Goeke, and E. Giniger. Lola regulates midline crossing of cns axons in drosophila. *Development*, 129(6):1317–25.

[9] M. P. Garcia and E. Becona. Evaluation of the amount of therapist contact in a smoking cessation program. *Span J Psychol*, 3(1):28–36.

[10] T. Hummel, K. Schimmelpfeng, and C. Klambt. Commissure formation in the embryonic cns of drosophila. *Dev Biol*, 209(2):381–98.

[11] J. Jack and G. Myette. The genes raw and ribbon are required for proper shape of tubular epithelial tissues in drosophila. *Genetics*, 147(1):243–53.

[12] H. Jasper, V. Benes, A. Atzberger, S. Sauer, W. Ansorge, and D. Bohmann. A genomic switch at the transition from cell proliferation to terminal differentiation in the drosophila eye. *Dev Cell*, 3(4):511–21.

[13] N. Kaufmann, J. DeProto, R. Ranjan, H. Wan, and D. Van Vactor. Drosophila liprin-alpha and the receptor phosphatase dlar control synapse morphogenesis. *Neuron*, 34(1):27–38.

[14] S. Kawaguchi and Y. Zheng. Characterization of a drosophila centrosome protein cp309 that shares homology with kendrin and cg-nap. *Mol Biol Cell*, 15(1):37–45.

[15] A. A. Kiger, B. Baum, S. Jones, M. R. Jones, A. Coulson, C. Echeverri, and N. Perrimon. A functional genomic analysis of cell morphology using rna interference. *J Biol*, 2(4):27.

[16] T. Komiyama, W. A. Johnson, L. Luo, and G. S. Jefferis. From lineage to wiring specificity. pou domain transcription factors control precise connections of drosophila olfactory projection neurons. *Cell*, 112(2):157–67.

[17] J. Y. Lee, A. E. Bielawska, and L. M. Obeid. Regulation of cyclin-dependent kinase 2 activity by ceramide. *Exp Cell Res*, 261(2):303–11.

[18] K. Madden, D. Crowner, and E. Giniger. Lola has the properties of a master regulator of axon-target interaction for snb motor axons of drosophila. *Dev Biol*, 213(2):301–13.

[19] B. D. McCabe, S. Hom, H. Aberle, R. D. Fetter, G. Marques, T. E. Haerry, H. Wan, M. B. O'Connor, C. S. Goodman, and A. P. Haghighi. Highwire regulates presynaptic bmp signaling essential for synaptic growth. *Neuron*, 41(6):891–905.

[20] V. L. McGovern, C. A. Pacak, S. T. Sewell, M. L. Turski, and M. A. Seeger. A targeted gain of function screen in the embryonic cns of drosophila. *Mech Dev*, 120(10):1193–207.

[21] J. Morales, P. R. Hiesinger, A. J. Schroeder, K. Kume, P. Verstreken, F. R. Jackson, D. L. Nelson, and B. A. Hassan. Drosophila fragile x protein, dfxr, regulates neuronal morphology and function in the brain. *Neuron*, 34(6):961–72.

[22] A. Myat, P. Henry, V. McCabe, L. Flintoft, D. Rotin, and G. Tear. Drosophila nedd4, a ubiquitin ligase, is recruited by commissureless to control cell surface levels of the roundabout receptor. *Neuron*, 35(3):447–59.

[23] T. P. Newsome, S. Schmidt, G. Dietzl, K. Keleman, B. Asling, A. Debant, and B. J. Dickson. Trio combines with dock to regulate pak activity during photoreceptor axon pathfinding in drosophila. *Cell*, 101(3):283–94.

[24] S. Onel, L. Bolke, and C. Klambt. The drosophila arf6-gef schizo controls commissure formation by regulating slit. *Development*, 131(11):2587–94.

[25] S. N. Prokopenko, A. Brumby, L. O'Keefe, L. Prior, Y. He, R. Saint, and H. J. Bellen. A putative exchange factor for rho1 gtpase is required for initiation of cytokinesis in drosophila. *Genes Dev*, 13(17):2301–14.

[26] S. Rajagopalan, E. Nicolas, V. Vivancos, J. Berger, and B. J. Dickson. Crossing the midline: roles and regulation of robo receptors. *Neuron*, 28(3):767–77.

[27] J. E. Reuter, T. M. Nardine, A. Penton, P. Billuart, E. K. Scott, T. Usui, T. Uemura, and L. Luo. A mosaic genetic screen for genes necessary for drosophila mushroom body neuronal morphogenesis. *Development*, 130(6):1203–13.

[28] A. Salzberg, S. N. Prokopenko, Y. He, P. Tsai, M. Pal, P. Maroy, D. M. Glover, P. Deak, and H. J. Bellen. P-element insertion alleles of essential genes on the third chromosome of drosophila melanogaster: mutations affecting embryonic pns development. *Genetics*, 147(4):1723–41.

[29] H. Sink, E. J. Rehm, L. Richstone, Y. M. Bulls, and C. S. Goodman. sidestep encodes a target-derived attractant essential for motor axon guidance in drosophila. *Cell*, 105(1):57–67.

[30] R. K. Smith, P. M. Carroll, J. D. Allard, and M. A. Simon. Mask, a large ankyrin repeat and kh domain-containing protein involved in drosophila receptor tyrosine kinase signaling. *Development*, 129(1):71–82.

[31] W. G. Somers and R. Saint. A rhogef and rho family gtpase-activating protein complex links the contractile ring to cortical microtubules at the onset of cytokinesis. *Dev Cell*, 4(1):29–39.

[32] T. Tal, D. Vaizel-Ohayon, and E. D. Schejter. Conserved interactions with cytoskeletal but not signaling elements are an essential aspect of drosophila wasp function. *Dev Biol*, 243(2):260–71.

[33] J. R. Terman, T. Mao, R. J. Pasterkamp, H. H. Yu, and A. L. Kolodkin. Micals, a family of conserved flavoprotein oxidoreductases, function in plexin-mediated axonal repulsion. *Cell*, 109(7):887–900.

[34] P. Wai, B. Truong, and K. M. Bhat. Cell division genes promote asymmetric interaction between numb and notch in the drosophila cns. *Development*, 126(12):2759–70.

[35] F. Wang, K. Dumstrei, T. Haag, and V. Hartenstein. The role of decadherin during cellularization, germ layer formation and early neurogenesis in the drosophila embryo. *Dev Biol*, 270(2):350–63.

[36] J. A. Williams, H. S. Su, A. Bernards, J. Field, and A. Sehgal. A circadian output in drosophila mediated by neurofibromatosis-1 and ras/mapk. *Science*, 293(5538):2251–6.

[37] X. Yang, L. Li, and X. A. Wang. [primary clinical experience of neuroendoscopy: report of 19 cases]. *Zhonghua Wai Ke Za Zhi*, 35(4):234–6.

[38] J. C. Yin, J. S. Wallach, M. Del Vecchio, E. L. Wilder, H. Zhou, W. G. Quinn, and T. Tully. Induction of a dominant negative creb transgene specifically blocks long-term memory in drosophila. *Cell*, 79(1):49–58.

[39] J. A. Zallen, Y. Cohen, A. M. Hudson, L. Cooley, E. Wieschaus, and E. D. Schejter. Scar is a primary regulator of arp2/3-dependent morphological events in drosophila. *J Cell Biol*, 156(4):689–701.

[40] L. Zheng, J. Zhang, and R. W. Carthew. frizzled regulates mirror-symmetric pattern formation in the drosophila eye. *Development*, 121(9):3045–55.
