# Supplementary material for: Transcriptional control in embryonic Drosophila midline guidance assessed through a whole genome approach
Source: BMC Neurosci. 2007 Jul 31;8:59. doi: 10.1186/1471-2202-8-59 (PMC1950096; doi:10.1186/1471-2202-8-59)
Supplement: Additional file 3 — Time Course Cluster 91. Annotations and In situ images for genes in Cluster TC91. [file 1471-2202-8-59-S3.pdf]

Table 1: Relevant Genes in Cluster 91

Symbol	Notes	BRP	Interactions	Reference
comm	axon guidance; neurogenesis; expr in VNC; phe axon guidance	0.37	ena, robo	[35]
robo2	axon guidance; VNC dev; phe axon guidance	0.47	Abl, ena, robo, slit	[36] [30]
comm2	axon guidance	0.65	robo	[18]
spi	trachea dev; phe commissure, glial cells; ligand Egfr	0.47		[10] [3]
hh	glial cell migration; phe commissure	0.69		[14]
argos	axon guidance; expr in CNS; phe axon guidance; photoreceptor guidance; eye dev	0.39	Egfr, Dl	[7] [34]
gsb	VNC dev; tf; phe commissures	0.34	slp2	[35] [5]
Dl	Notch signaling; Notch binding; adhesion, VNC dev, neurogenesis; phe fascicle, axon guidance; expr in VNC	0.66	argos, Brd, neur, kuz	[9]
slp2	phe RP2 neuron; expr CNS; tf	0.45	gsb	[5]
siz	guanyl-nucleotide exchange factor activity; phe commissures	0.33	slit, fra	[26]
ems	tf; trachea dev; expr in CNS; phe axon guidance	0.74		[11]
RhoGEF4	RhoGEF; regulation of cell shape	0.34		[19]
RhoGEF3	RhoGEF	0.89		[13]
CG32560	Ras GTPase activator	0.41		
Thor	initiation factor; expr in CNS; immunity	0.34		
CG11597	protein phosphatase type 2A	0.55		
BEST:LD13681	cytoskeleton	0.33		
eya	tyrosine phosphatase; Bolwigs organ morphogenesis; eye dev; neuronal dev	0.58	ato	[28] [37]
Ly	tf; PNS dev; eye dev; phe PNS, R8 receptor	0.48		
vihar	ubiquitin-conjugating	0.59		[23]
ppa	ubiquitin	0.35		[29]
CG11136	cell adhesion	0.36		
ato	tf; neurogenesis; brain dev; phe glial cell	0.54		[12] [16]
btl	glia cell migration; trachea dev; expr in ventral midline; phe glia cell migration	0.54		[15]
nub	tf; VNC dev; expr in CNS; phe RP1-4 neurons	0.49		[38]
ase	tf; CNS dev; neurogenesis; expr in CNS	1		[27]
King-tubby	expr in CNS	0.75		[32]
phyl	PNS dev; R7 dev	0.78		[8]
bib	Notch; neurogenesis; expr in ventral midline, CNS; phe CNS	0.70		[31]
HLHmgamma	tf; Notch signaling; neurogenesis; E(spl) complex; phe CNS	0.37		[25]
HLHm5	tf; Notch signaling; neurogenesis; E(spl) complex; phe CNS	0.90		[25]

Continued on next page

Symbol	Notes	BRP	Interactions	Reference
m4	Notch signaling; E(spl) complex; phe CNS	0.89	$m\alpha$	[24]
Brd	Notch signaling	0.74	neur	[21]
neur	neu; Notch signaling; PNS dev; phe CNS	0.46	Brd	[22]
E(spl)	Notch signaling; PNS dev; neurogenesis	0.53	Dl	[20]
mα	Notch signaling; E(spl) complex; sensory organ dev	0.48	m4	[1]

Note: phe – phenotype; expr – expressed; MB – Mushroom Bodies; dev – development; NMJ – Neuromuscular Junction; tf – transcription factor

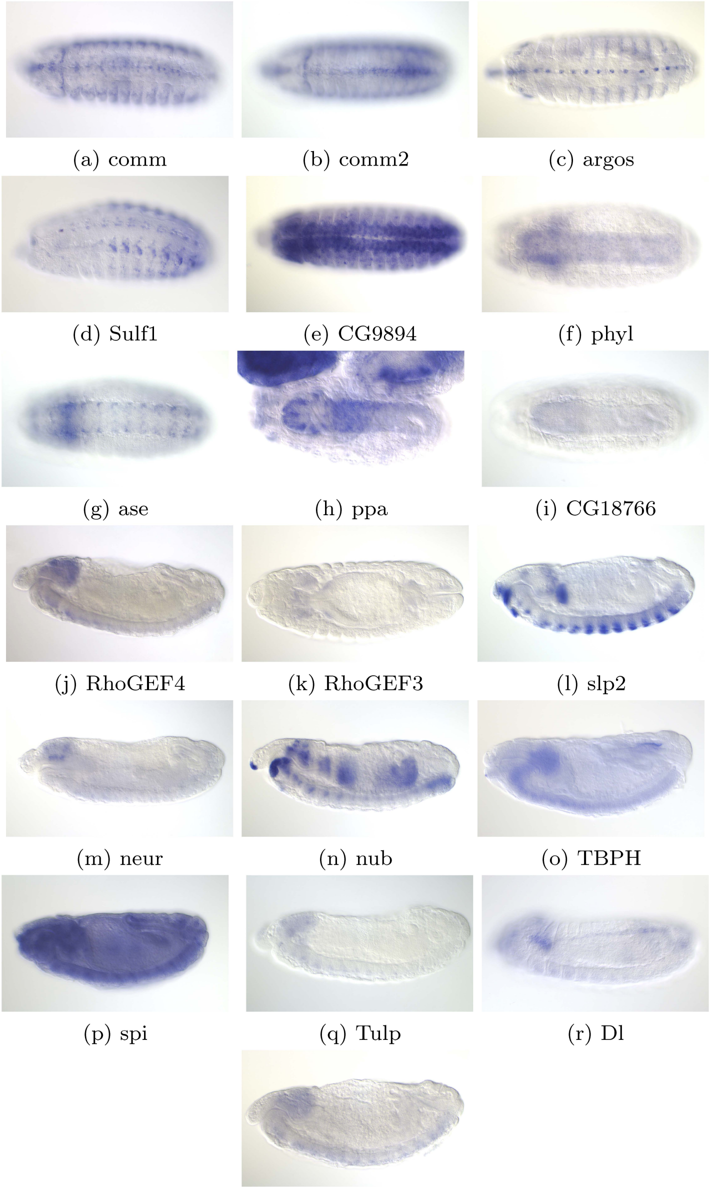

(a) comm (b) comm2 (c) argos
(d) Sulf1 (e) CG9894 (f) phyl
(g) ase (h) ppa (i) CG18766
(j) RhoGEF4 (k) RhoGEF3 (l) slp2
(m) neur (n) nub (o) TBPH
(p) spi (q) Tulp (r) Dl
(s) CG13434

Figure 1: **APoGE In Situ Hybridization for Cluster 91**

Figure 1: **APoGE In Situ Hybridization for Cluster 91 (cont.)** APoGE was queried for clusters 87 to 97, comprising 164 genes, of which 84 are annotated in APoGE. (1a) to (1i) are shown ventrally. (1a) to (1d) are expressed in the midline. (1j), and (1l) to 1s) are shown laterally. (1k) is shown dorsally. (1e) to (1s) are genes expressed in the VNC and embryonic brain, as seen in the picture and as annotated in APoGE. (1a) comm 1s) (1b) comm2 are expressed at the midline. (1c) Argos has a midline expression similar to comm and comm2; Argos is involved in axon guidance in the visual system [7]. (1d) Sulf1 midline pattern is apparently adjacent to comm, comm2 and argos; Sulf1 has been implicated in the patterning of the cuticle, but one reference links Sulf1 to the midline [17]. (1e) CG9894 is an unknown gene, placed in Cluster 91 immediately after comm, robo2, spit and BEST:LD13681. (1f) phyl is involved in cell fate determination of photoreceptors and nervous system development [8]. (1g) ase is a neural precursor gene involved in neurogenesis [6]. (1h) ppa, (1i) CG18766, (1j) RhoGEF4 and (1k) RhoGEF3 are members of the family of Rho guanyl-nucleotide exchange factor activity family for which other members have established roles in axon guidance [2]. (1l) slp2 is involved in period partitioning. (1m) neur is involved in peripheral neurogenesis [33]. (1n) nub is involved in neurogenesis and neurospecification [4]. (1o) TBHP is involved in RNA splicing. (1p) spi is part of the epidermal growth factor receptor signaling pathway. (1q) king tubby is relevant in olfactory perception. (1r) Dl is part of the Notch signaling and is involved in Notch binding. (1s) CG13434 is an unknown gene with CNS expression.

References

[1] Y. Apidianakis, A. C. Nagel, A. Chalkiadaki, A. Preiss, and C. Delidakis. Overexpression of the m4 and malpha genes of the e(spl)-complex antagonizes notch mediated lateral inhibition. *Mech Dev*, 86(1-2):39–50.

[2] G. J. Bashaw, H. Hu, C. D. Nobes, and C. S. Goodman. A novel dbl family rhogef promotes rho-dependent axon attraction to the central nervous system midline in drosophila and overcomes robo repulsion. *J Cell Biol*, 155(7):1117–22.

[3] A. Bergmann, M. Tugentman, B. Z. Shilo, and H. Steller. Regulation of cell number by mapk-dependent control of apoptosis: a mechanism for trophic survival signaling. *Dev Cell*, 2(2):159–70.

[4] K. M. Bhat, S. J. Poole, and P. Schedl. The miti-mere and pdm1 genes collaborate during specification of the rp2/sib lineage in drosophila neurogenesis. *Mol Cell Biol*, 15(8):4052–63.

[5] K. M. Bhat, E. H. van Beers, and P. Bhat. Sloppy paired acts as the downstream target of wingless in the drosophila cns and interaction between sloppy paired and gooseberry inhibits sloppy paired during neurogenesis. *Development*, 127(3):655–65.

[6] M. Brand, A. P. Jarman, L. Y. Jan, and Y. N. Jan. asense is a drosophila neural precursor gene and is capable of initiating sense organ formation. *Development*, 119(1):1–17.

[7] A. Brunner, T. Twardzik, and S. Schneuwly. The drosophila giant lens gene plays a dual role in eye and optic lobe development: inhibition of differentiation of ommatidial cells and interference in photoreceptor axon guidance. *Mech Dev*, 48(3):175–85.

[8] H. C. Chang, N. M. Solomon, D. A. Wassarman, F. D. Karim, M. Therrien, G. M. Rubin, and T. Wolff. phyllopod functions in the fate determination of a subset of photoreceptors in drosophila. *Cell*, 80(3):463–72.

[9] D. Crowner, M. Le Gall, M. A. Gates, and E. Giniger. Notch steers drosophila isnb motor axons by regulating the abl signaling pathway. *Curr Biol*, 13(11):967–72.

[10] P. Duchek and P. Rorth. Guidance of cell migration by egf receptor signaling during drosophila oogenesis. *Science*, 291(5501):131–3.

[11] B. Hartmann, F. Hirth, U. Walldorf, and H. Reichert. Expression, regulation and function of the homeobox gene empty spiracles in brain and ventral nerve cord development of drosophila. *Mech Dev*, 90(2):143–53.

[12] B. A. Hassan, N. A. Bermingham, Y. He, Y. Sun, Y. N. Jan, H. Y. Zoghbi, and H. J. Bellen. atonal regulates neurite arborization but does not act as a proneural gene in the drosophila brain. *Neuron*, 25(3):549–61.

[13] M. S. Hicks, V. O'Leary, M. Wilkin, S. E. Bee, M. J. Humphries, and M. Baron. Drhogef3 encodes a new drosophila dh domain protein that exhibits a highly dynamic embryonic expression pattern. *Dev Genes Evol*, 211(5):263–7.

[14] T. Hummel, S. Attix, D. Gunning, and S. L. Zipursky. Temporal control of glial cell migration in the drosophila eye requires gilgamesh, hedgehog, and eye specification genes. *Neuron*, 33(2):193–203.

[15] J. Jarecki, E. Johnson, and M. A. Krasnow. Oxygen regulation of airway branching in drosophila is mediated by branchless fgf. *Cell*, 99(2):211–20.

[16] D. Jhaveri, A. Sen, and V. Rodrigues. Mechanisms underlying olfactory neuronal connectivity in drosophila-the atonal lineage organizes the periphery while sensory neurons and glia pattern the olfactory lobe. *Dev Biol*, 226(1):73–87.

[17] J. B. Kearney, S. R. Wheeler, P. Estes, B. Parente, and S. T. Crews. Gene expression profiling of the developing drosophila cns midline cells. *Dev Biol*, 275(2):473–92.

[18] K. Keleman, S. Rajagopalan, D. Cleppien, D. Teis, K. Paiha, L. A. Huber, G. M. Technau, and B. J. Dickson. Comm sorts robo to control axon guidance at the drosophila midline. *Cell*, 110(4):415–27.

[19] A. A. Kiger, B. Baum, S. Jones, M. R. Jones, A. Coulson, C. Echeverri, and N. Perrimon. A functional genomic analysis of cell morphology using rna interference. *J Biol*, 2(4):27.

[20] E. Knust, K. A. Bremer, H. Vassin, A. Ziemer, U. Tepass, and J. A. Campos-Ortega. The enhancer of split locus and neurogenesis in drosophila melanogaster. *Dev Biol*, 122(1):262–73.

[21] E. C. Lai, R. Bodner, J. Kavaler, G. Freschi, and J. W. Posakony. Antagonism of notch signaling activity by members of a novel protein family encoded by the bearded and enhancer of split gene complexes. *Development*, 127(2):291–306.

[22] E. C. Lai, G. A. Deblandre, C. Kintner, and G. M. Rubin. Drosophila neuralized is a ubiquitin ligase that promotes the internalization and degradation of delta. *Dev Cell*, 1(6):783–94.

[23] E. Mathe, C. Kraft, R. Giet, P. Deak, J. M. Peters, and D. M. Glover. The e2-c vihar is required for the correct spatiotemporal proteolysis of cyclin b and itself undergoes cyclical degradation. *Curr Biol*, 14(19):1723–33.

[24] A. C. Nagel, Y. Apidianakis, I. Wech, D. Maier, C. Delidakis, and A. Preiss. Neural hyperplasia induced by rna interference with m4/malpha gene activity. *Mech Dev*, 98(1-2):19–28.

[25] K. Nakao and J. A. Campos-Ortega. Persistent expression of genes of the enhancer of split complex suppresses neural development in drosophila. *Neuron*, 16(2):275–86.

[26] S. Onel, L. Bolke, and C. Klambt. The drosophila arf6-gef schizo controls commissure formation by regulating slit. *Development*, 131(11):2587–94.

[27] C. Parras, L. A. Garcia-Alonso, I. Rodriguez, and F. Jimenez. Control of neural precursor specification by proneural proteins in the cns of drosophila. *Embo J*, 15(23):6394–9.

[28] F. Pignoni, B. Hu, K. H. Zavitz, J. Xiao, P. A. Garrity, and S. L. Zipursky. The eye-specification proteins so and eya form a complex and regulate multiple steps in drosophila eye development. *Cell*, 91(7):881–91.

[29] L. Raj, P. Vivekanand, T. K. Das, E. Badam, M. Fernandes, R. L. Finley, R. Brent, L. F. Appel, S. D. Hanes, and M. Weir. Targeted localized degradation of paired protein in drosophila development. *Curr Biol*, 10(20):1265–72.

[30] S. Rajagopalan, E. Nicolas, V. Vivancos, J. Berger, and B. J. Dickson. Crossing the midline: roles and regulation of robo receptors. *Neuron*, 28(3):767–77.

[31] Y. Rao, R. Bodmer, L. Y. Jan, and Y. N. Jan. The big brain gene of drosophila functions to control the number of neuronal precursors in the peripheral nervous system. *Development*, 116(1):31–40.

[32] M. Ronshaugen, N. McGinnis, D. Inglis, D. Chou, J. Zhao, and W. McGinnis. Structure and expression patterns of drosophila tulp and tusp, members of the tubby-like gene family. *Mech Dev*, 117(1-2):209–15.

[33] A. Salzberg, D. D'Evelyn, K. L. Schulze, J. K. Lee, D. Strumpf, L. Tsai, and H. J. Bellen. Mutations affecting the pattern of the pns in drosophila reveal novel aspects of neuronal development. *Neuron*, 13(2):269–87.

[34] K. Sawamoto, M. Okabe, T. Tanimura, S. Hayashi, K. Mikoshiba, and H. Okano. argos is required for projection of photoreceptor axons during optic lobe development in drosophila. *Dev Dyn*, 205(2):162–71.

[35] M. Seeger, G. Tear, D. Ferres-Marco, and C. S. Goodman. Mutations affecting growth cone guidance in drosophila: genes necessary for guidance toward or away from the midline. *Neuron*, 10(3):409–26.

[36] J. H. Simpson, T. Kidd, K. S. Bland, and C. S. Goodman. Short-range and long-range guidance by slit and its robo receptors. robo and robo2 play distinct roles in midline guidance. *Neuron*, 28(3):753–66.

[37] T. Suzuki and K. Saigo. Transcriptional regulation of atonal required for drosophila larval eye development by concerted action of eyes absent, sine oculis and hedgehog signaling independent of fused kinase and cubitus interruptus. *Development*, 127(7):1531–40.

[38] S. L. Yeo, A. Lloyd, K. Kozak, A. Dinh, T. Dick, X. Yang, S. Sakonju, and W. Chia. On the functional overlap between two drosophila pou homeo domain genes and the cell fate specification of a cns neural precursor. *Genes Dev*, 9(10):1223–36.
